# Progress and Perspectives on Ceramic Membranes for Solvent Recovery

**DOI:** 10.3390/membranes9100128

**Published:** 2019-10-04

**Authors:** Senthilnathan Ruthusree, Subramanian Sundarrajan, Seeram Ramakrishna

**Affiliations:** Center for Nanofibers and Nanotechnology Lab, Mechanical Engineering, National University of Singapore, Blk E3 05-12, 2 Engineering Drive 3, Singapore 117581, Singapore; ruthusree6@gmail.com

**Keywords:** solvent dehydration, solvent recovery, pervaporation

## Abstract

With the increase in demand for commodities in the world, it is advisable to conserve resources. In the case of liquid wastes generated from pharmaceutical and petroleum industries, an unconventional solution is provided for the regeneration of solvents. However, this solvent recovery can be carried out using various efficient methods. Recently, Mixed Matrix Membranes (MMM) obtained by the addition of nanoparticles into a polymer matrix as reinforcements, or using a material with a well-defined inorganic network as a membrane like zeolite, silica based, Zeolite imidazolate frameworks (ZIFs) and Metal organic frameworks (MOFs), were explored for a solvent recovery process. These membranes possess characteristics such as high selectivity, flux and stability at various environmental conditions for the solvent recovery process. In this review, we have covered the polymer, nanocomposites, and ceramic membranes for solvent recovery through the pervaporation and organic solvent nanofiltration processes. The key challenges faced by the materials such as MOFs, zeolite, silica, zeolite and ZIFs when they are fabricated (through in situ synthesis or secondary growth process) as membranes and separation of solvents to explore for the solvent recovery process are reviewed.

## 1. Introduction

Solvents find their applications in an ample variety of pharmaceutical and other industrial products. The liquid wastes generated from the manufacturers can be hazardous, making their disposal a costly affair [1,2,3,4,5]. Storage of the waste occupies plethora of workspace and, in addition results in occupational hazard. When the waste generated is on a small scale, disposal can be easy; however, an alternative solution of recycling the liquid discharge can be considered. In order to fulfill the demands of living things, conserving and recycling of sources would be an easier, greener- and more economical approach. Recycling of solvent effluent waste can help to the recover the valuable solvents, termed as solvent recovery. Solvent recovery can considerably not only reduce the waste generation, but also the transport cost, especially when the solvents are expensive and large volumes are involved. The recycling of solvent waste can dramatically bring down the cost of solvents by 80%, with their ability to recycle up to 95% of waste and using them for 10 cycles. 

Solvent recycling is typically used in manufacturing units generating wastes such as pharmaceutical industry, paint lacquer, metal finishing and automotive coatings. This recycling concept has been implemented in industry for many decades and is a routine practice when it comes to the pharmaceutical industry; they serve as an economically viable solution for the regeneration of a particular solvent waste [6,7]. Recovery of solvents from the effluent can be carried out in various processes or techniques, subjected to size of the batch and also the automation [8]. This technique has limitations such as high initial cost to setup the process, technological barrier and process complexity. The traditional method used for recycling is fractional distillation, but, with the advances in techniques, liquid–liquid extraction, absorbing systems, film evaporation, crystallization and separation process using membranes can be used for the particular application [9]. The liquid waste that can be treated can be: (1) non-azeotropic solution, (2) aqueous non azeotropic solution, (3) heterogeneous azeotropic solution, or (4) aqueous homogeneous azeotropic solution.

Recently, membrane technology has found their widespread adoption in the solvent recovery process due to their improved efficiency and low energy cost [10]. The membranes used in this process for solvent recovery are generally made of either organic or inorganic materials. Organic materials contribute the polymeric membranes which are both natural and synthetic. The inorganic ones consist of artificial ceramic, metallic and zeolite membranes [11]. Ceramic membranes consist of materials like alumina and titania; they also contain compounds like carbides and nitrides. Due to their inertness, they are used for highly acidic or basic environments [11]. They fail due to the highly sensitive temperature gradient, which leads to the cracking of membrane. Zeolite membranes are synthesized hydrothermally on to a porous ceramic or metal support in the presence of heat [12]. They also require a thicker layer to protect them from the formation of cracks and pinholes. Inorganic membranes possess few advantages like high thermal and chemical stabilities, inertness to microbiological degradation and reduction in cleaning requirements [13]. 

The membrane is fabricated on a porous support to improve the permeability. The establishment of a selective layer on a porous support enhances the permeability and selectivity of the membrane. Despite its advantages, they face complications arising because of the fall in the thermal compatibility between the porous support and the selective layer [14]. Coefficient of thermal expansion in metal oxides and membranes play a major role in the material selection criteria [10].

Although the process of solvent recovery is very advantageous in preserving and conserving the waste solvent, it has obstacles to overcome in order to be explored in real application. The major drawback impeding this process is the existence of azeotropic compositions in the liquid discharge. Azeotrope is a mixture having the same composition in both liquid and vapor phase. Hence, the separation of the mixture via fractional distillation was a failure. Alternative solutions for resolving the hurdle are azeotropic distillation, molecular sieve and membranes for azeotropic mixture separation [13]. In the case of azeotropic distillation, there is an addition of a third component to the mixture to generate a contemporary lower body azeotrope, which is heterogeneous and forms a separate phase for extractive distillation. Molecular sieve absorbents have pores of uniform size which are identical to the dimensions of small molecules; they also restrict the entry of large molecules and, with heat treatment, the sieves can be regenerated [15].

Although reviews have already been published in the concept of solvent recovery, they are focused on the general aspects [4,16,17,18,19]. The study conducted by Yao et al. [16] has highlighted the synthesis of Zeolitic imidazolate framework (ZIF) membranes, thin film formation and their application as pervaporation membranes for dehydration (till the year 2014). The review by Betard et al. [17] has stated some views on the fundamental concepts and applications of MOF thin films, such as sensing and gas separation till 2012. The purification and recovery of solvent waste in the manufacture of active pharmaceutical ingredients (API’s) and three case studies are presented by Cavanagh et al. [4]. To date, there has not been any solid comprehensive review that reports the progress in the aspects of solvent recovery by membranes. In fact, the most recent developments have been published over the past few years.

Lately, the synthesis of continuous ZIF-68 membranes on alumina support by reactive seeding was developed by Kasik et al. [18]. More recently, ZIFs with higher pore opening materials (largest pore opening of 22.5 Å and the largest cage size of 45.8 Å) were synthesized and tested for the removal of organic mixtures such as octane and p-xylene from humid air [19]. Hence, in this review, mixed matrix membrane, Zeolite based membranes, Mordenite Framework Inverted (MFI) Type Zeolite, metal organic frameworks (MOF), silica-based membranes and Zeolite imidazolate framework (ZIF) for the separation of organic solvents from their corresponding solvent mixture (or water) is highlighted. 

## 2. Inorganic Membranes

### 2.1. Zeolite-Based Membranes

**Figure 1:**
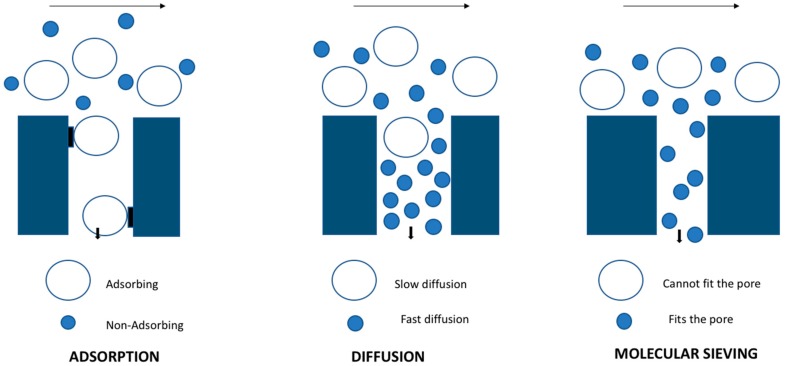
Separation mechanism in zeolite membrane.

Zeolites are artificially or naturally occurring materials with a uniform and well-defined network of pores. They are crystalline in nature and are composed of materials like silicon, aluminum and oxygen, as well as other cations required for the framework. They have pore sizes varying from 0.3 to 1.3 nm, depending on the type of framework. In case of the synthetic zeolite, the synthesis procedure determines the ratio of materials used (Si/Al), and it simultaneously affects the material properties such as polarity and adsorption. Zeolite membranes are inorganic in nature and have inter grown zeolite crystals on the porous membranes. They have incredible properties like high thermal and chemical stabilities to stand over polymeric membranes, and also possess high flux and selectivity through the well-defined pore size (Figure 1). During a pervaporation process, the zeolitic and non-zeolitic pores of the Linde type A Sodium (zeolite NaA) membranes strongly adsorb the water vapor and undergo condensation. As a result, the water permeates with high permeance though the capillary condensation mechanism and hinders other molecules permeation by obstructing their entry into the pores. Zeolite membranes have found their application on a pharmaceutical industrial scale for solvent dehydration. Another factor determining the productivity of the membranes are thickness and defects found in them. The permeability of solvents can be diminished with the high film thickness and selectivity due to defects. Hedlund et al. [20] reported that, in order to obtain high flux, the thickness of membrane should be 1 μm and ideally supported by a substrate. Zeolite films on substrate can be fabricated using in situ synthesis and secondary growth with hydrothermal treatment. Many recent publications show that the secondary growth method favors high reproducibility and good control over the membrane structure [18,21].

LTA (Linde type A) zeolite membranes are the first membranes introduced commercially in the market for commercial use. They have a three-dimensional porous structure with pores running perpendicular to all the planes. Zeolite A is synthesized using a sol-gel technique by the reaction of alumina and silica in an alkaline medium. The super cage structure possessed by the zeolite makes the membrane enhance both the selectivity and flux. The presence of alumina in the membrane improves the hydrophilicity of the system.

Zeolite membranes were commercially first developed by the Mitsui Engineering and Shipbuilding Co. (Tokyo, Japan) [11]. In association with Yamaguchi University, for the purpose of organic solvent degradation in an expansive scale, they fabricated the zeolite membrane on the surface of ceramic alumina tube of 1.2 cm OD and 80 cm length for ethanol and solvent dehydration (10 wt% of water content) and tested separation performance by pervaporation experiment The synthesized membranes exhibited high flux rates at different temperatures (water/isopropanol (up to 16 kg/(m^2^·h), 120 °C), water/ethanol (4.5 kg/(m^2^·h), 105 °С) and water/dioxane (7.8 kg/(m^2^·h), 105 °С)) indicating that temperature plays a vital role in governing the permeate flux. The separation factor of more than 1000 was observed, indicating that high selectivity of the separation can be achieved. However, the membranes showed enhancement in solvent flux, but the usage of tubular zeolite membranes seems to be costly [11]. To overcome this issue, Zhang et al. [22] introduced thin, porous metal sheets to reinforce the zeolite membrane. The fabrication of these high flux and selective zeolite NaA membrane is formed from the porous metal sheets along with the two-time dip coating–wiping seed deposition procedure. The substrate used has a smooth and uniform porous structure over the surface. Apart from this, the porous metal sheets provide beneficial properties such as high permeability, chemical stability, mechanical strength, membrane packing density and low cost. With increase in separation temperature during the pervaporation process in the setup, the selectivity and permeability of solvents also increase gradually. With an elevation in the separation temperature from 75 °C to 135 °C, there was a uniform increase in the water/ethanol permeation flux and selectivity. The water/ethanol selectivity fluctuated between 10,000 and 70,000 and displayed water permeation flux of above 4kg/(m^2^·h) starting at 75 °C with a feed of 10% (w/w) water in ethanol. The selectivity of water/ethanol improved with an increase in water content. However, these membranes fail to possess enough selectivity with high fluxes for potential industrial application. To counter this problem, Mastropietro et al. [23] modified a procedure for simple synthesis of FAU membranes composed by hierarchically assembled Nano-zeolites. Initially, these membranes demonstrated high water vapor fluxes as well as high selectivity values for water vapor, which are mostly due to the higher rate of diffusion for water with respect to the N_2_ molecular probe within the hydrophilic zeolite network. Therefore, the size of crystallites of the membrane was reduced to enhance the affinity of water which leads to stronger interactions with water molecules and permeation by approximately 5 times (8 to 39.9 μmole·m^−2^·s^−1^·Pa^−1^) times at the steady state. The pervaporation permeation of different organic/water liquid mixtures studied by Okamoto et al. is tabulated in Table 1 [24]. 

The membrane pores are smaller than the size of the small organic molecules for the separation of mixtures. The diffusion rates of water, 2-propanol, methanol, ethanol and acetone through a Ge-ZSM 5 zeolite membrane by using isotopic- transient pervaporation were measured by Bowen et al. [25]. In the methanol/ethanol mixtures, the presence of methanol increased the rate of the diffusion of ethanol. In the feed of methanol/ethanol mixtures containing 95 wt% of ethanol at 313 K, the fluxes reported for ethanol and methanol are 3.8 and 0.2 mol/m^2^·h, respectively.

**Figure 2:**
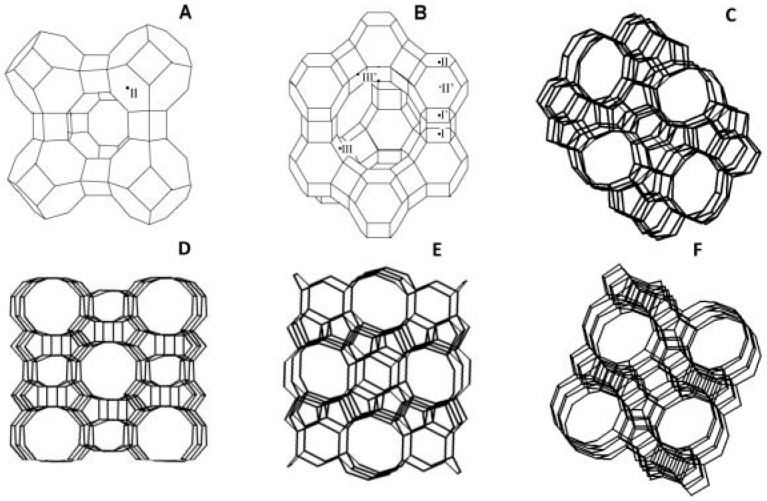
Industrially most important zeolite structures: (**A**) LTA; (**B**) FAU; (**C**) ZSM-5 (Zeolite Socony Mobil-5); (**D**) MOR (Mordenite Framework); (**E**) FER (Ferrierite); and (**F**) BEA (Beta Polymorph). The lines represent the O atoms and the corners Si or Al atoms, and the exchangeable cation sites in LTA and FAU are indicated; Reproduced with permission from [29].

The separation of permeating molecules through zeolite membranes takes place because of the difference in chemical affinities, along with the shape and size with respect to the pores. MFI-type zeolite is a high silica zeolite synthesized using hydroxide of tetra propylammonium as a template. This avenue of research played a remarkable role in the petrochemical industry. For the separation of liquid mixture through pervaporation process, a liquid feed is used and the permeate is a vapor. It is also reported that, with an increase in partial pressure of feeds, the separation factor decreases. In the pervaporation experiments conducted by Algieri et al. [26], thin MFI zeolite membranes were synthesized by in situ nucleation and secondary growth at 70 °C. They fed 9.4 wt% of ethanol–water mixtures and high fluxes (2.1 kg/m^2^·h), and separation factor as low as 1.3 were obtained. They also reported fluxes and separation factor for NaA type zeolite membrane varying from 0.23 to 5.60 kg/m^2^·h and 3600 to 10,000, respectively. Kanezashi et al. [27] reported the usage of MFI type zeolite membrane for the pervaporation of p- and o-xylene binary mixture. At 25 °C and 1.1 kPa partial pressure, the obtained p-xylene flux and p/o-xylene selectivity were 7.6 × 10^−4^ and 22 mol/m^2^·s, respectively. Wang et al. [28] reported the utilization of MFI Zeolite membranes for the separation of CO_2_/Xe gas mixtures with a separation factor of 5.6, and separating CO_2_ molecules of 3.3 Å from Xe molecules of 4.1 Å. The most important industrially explored zeolite structures are given in Figure 2 [29]. 

Kumakiri et al. [30] studied the FAU zeolite membranes to selectively separate the methanol solvent from various feed mixtures such as methanol–MMA–butanol–BMA. When a mixture of 20.2/29.7/9.2/40.9 wt% of butanol–MMA–methanol–BMA, respectively, was fed at 60 °C through a FAU zeolite membrane, the permeate had a composition of 1.60/0.17/98.1/0.17 wt% of butanol–MMA–methanol–BMA, respectively. This study showed that methanol selectivity can be achieved through FAU zeolite membrane in a quaternary mixture with total flux of about 2.0 kg·m^−2^·h^−1^. 

Dip coating, rubbing, and reactive seeding are some of the methods that are applied effectively for seeding the outer surface of the support material [31,32,33]. In the zeolite membrane synthesis, rubbing alone or a rubbing-dip coating technique were applied for seeding the zeolite on support materials such as α-alumina and secondary growth followed by pervaporation study by various authors. Applying the rubbing method, for the ethanol/water (90%) as feed, the obtained separation factors and fluxes were 3603, >10,000, >10,000 and 3.8, 3.17, 3.6 kg·m^−2^·h^−1^, respectively, by Pina, Ma and Wang et al. [31,32,33]. Similarly, when the rubbing-dip coating method was applied, for the ethylene glycol/water system as feed, the obtained separation factors and fluxes at 80 °C were 10,996, 7.16 kg·m^−2^·h^−1^, respectively by Jafari et al. [34], and for ethanol/water (90%) as feed at 70 °C, the obtained separation factors and fluxes were >15,000, 3.26 kg·m^−2^·h^−1^, respectively, by Liu et al. [35]. 

An ensemble synthesis strategy was applied on hollow fiber supported T-type zeolite membrane modules by the secondary growth method by Ji et al. [36]. Optimization of seed particles size, seed concentration, coating time and crystallization time were studied. The high-quality membrane modules with membrane areas of 0.03 m^2^ showed an average flux of 2.25 kg·m^−2^·h^−1^ and separation factor of 1348 for the dehydration of 90 wt% ethanol/water solutions at 348 K, whereas a pilot-scale apparatus of two 0.54 m^2^ membrane modules connected serially showed pervaporation dehydration of 90 wt% isopropanol/water mixture to 99.3 wt%.

Two different hydrophilic topologies of zeolites such as Faujasite (FAU) and mordenite (MOR) were investigated as membrane layers on tubular mullite and disk-shaped α-alumina supports for PV dehydration of ethanol by Asghari et al. [37]. Various synthesis parameters such as ceramic support, repetition of coating, seeding method, crystallization time (14 and 18 h), temperature (160, 170 and 180 °C), and Si/Al ratio of the precursor gel formulation (12 and 16) on the membranes structures and their PV performances were studied. Permeation flux (43.43%), separation factor (30.39%) and PSI (62.35%) decrease with increasing ethanol concentration in feed (50–90 wt%) was observed and the MOR layer on α-alumina (MOR_D-II) was reported as the best membrane for ethanol/water PV system. NaA zeolite pervaporation membranes was synthesized on the alumina hollow fiber inner-surface in a continuous flow system by Cao et al. [38]. The optimal PV performance of up to 19.7 kg/m^2^·h for the permeation flux and more than 80,000 for the separation factor at the flow rate of 148 mL/min with 90 wt% ethanol/water solutions at 348 K was reached and yielded an unprecedented performance. High-quality hollow fiber supported Decadodecasil 3R zeolite membranes were prepared on four-channel ceramic hollow fibers and successfully tested in pervaporation dehydration of acetic acid (AcOH) for the first time by Zhang et al. [39]. The membrane showed a water permeation flux of 0.58 kg·m^−2^·h^−1^ and a separation factor of 800 for the dehydration of 70 wt% water/AcOH mixture at 368 K and the stable permeation flux and separation factor was observed even in the presence of inorganic acid, thereby showing outstanding acid resistance.

Although various developments in the zeolite synthesis were achieved by giving importance to techno-economic criteria with an aim of industrial scaling, the environmental impact of these processes has not been studied. Normally, aggressive solvents were used, which has resulted in the environmental degradation. A life cycle assessment (LCA) tool has been most widely used to evaluate the environmental impacts of the manufacturing processes, which has been used in gas separation, water treatment, and alcohol purification by pervaporation. Navajas et al. [40] applied the LCA for the first time to zeolite membrane synthesis and quantified the effect of: (i) seed layers that allow membranes of submicron thickness; (ii) gel-less secondary treatments that avoid the use of large amounts of expensive structure directing agents; and (iii) use of low-cost polymer supports instead of conventional ceramic supports using GaBi 8.7 Pro software. They found that most of the impacts were given by the support layer and progresses in the synthesis of hollow fibers (thinner fibers) and use of less-aggressive solvents could significantly reduce the environmental impacts related with overall membrane synthesis.

The cost of the zeolite membranes are about ten times more expensive than polymeric membranes [41]. Although the inorganic membranes are costlier, their longer lifetime, high water concentration operating range and high thermal stability are added advantages when compared to the polymeric counterparts. In the early stages of zeolite membranes development, in addition to cost, the membranes developed were of low water flux due to thicker zeolite layers required and low water selectivity due to grain boundary/intercrystalline defects. However, these disadvantages were overcome by the deposition of a thin and uniform layer of zeolite seed crystals onto the porous support followed by secondary crystal growth treatment, which resulted in a thinner defect-free zeolite layer [42,43]. 

### 2.2. Silica-Based Membranes

Silica has the ability to link together with different amorphous or crystalline solids, to give rise to porous structures. The pore sizes of the material can be fine-tuned by using surfactant micelles (cetyltrimethylammonium bromide (CTAB) and sodium dodecyl sulfate (SDS)) to separate molecules based on their pore size with the property of size specificity through the pervaporation process. Elements like zirconia, alumina and titania are chosen as secondary elements for inclusion in the silica membrane to enhance the hydrothermal stability of membrane. The solvent used determines the rate of rejection. Silica membranes are generally casted using both chemical- and physical routes, where the sol-gel technique contributes to a chemical route and chemical vapour deposition to a physical one. Membranes are required to be used in non-aqueous system for the purpose of regenerating organic solvents. Tsuru et al. [44] made use of porous silica-zirconia membrane with nanopores in the range of 1–4 nm for the application of separating solvents like ethanol and methanol from non-aqueous solutions such as polyethylene glycol and ethylene glycol. 

Silica membranes were synthesized on α-alumina, γ-alumina, alumina, support by various researchers [45,46,47,48]. Silica MEL membrane was synthesized on a porous α-alumina hollow fiber support by a secondary growth approach. Kosinov et al. [45] have reported that silicalite-2 membrane showed higher fluxes without compromising selectivity for ethanol/water separation by pervaporation. However, only different selectivity was obtained for n-/i-butane mixture separation, in which MEL structure showed favorable diffusion to branched alkane compared to the MFI one due to the different pore topologies. It further showed that hydrophobic silicalite-2 membranes have the potential for the removal of organics from the aqueous solutions. Similarly, Boutikos et al. [47] applied silica membranes on γ-alumina for the separation of n-butanol and water mixture with a flux of 196 mol/m^3^.hr and separation factor of 150. Cobalt-doped silica was explored on α-alumina support for the separation of ethanol/water mixture with flux of 60 mol/m^3^·h and separation factor of 2530 by Wang et al. [49].

Hydrophobicity was introduced onto alumina and titania micro and meso porous ceramic membranes by grafting of C_6_F_13_C_2_H_4_Si(OEt)_3_ (C_6_) molecules by Kujawa et al. [50] and tested for the removal of hazardous volatile organic solvents (methyl tert-butyl-ether (MTBE), ethyl acetate (EtAc) and butanol (BuOH)) from binary aqueous solutions by the pervaporation process. They reported that the highest efficiency was achieved using a Titania membrane, which was characterized by the highest value of the pervaporation separation index (PSI) and the highest value of permeate flux of organic compounds in water–EtAc (J_EtAc_ = 1.1 kg·m^−2^·h^−1^; PSI_EtAc_ = 140 kg·m^−2^·h^−1^) and water–MTBE (J_MTBE_ = 1.0 kg·m^−2^·h^−1^; PSI_MTBE_ = 194 kg·m^−2^·h^−1^) systems. Tres et al. [51] have studied the potential applicability of ceramic membrane technology in vegetable oil processing and biodiesel industries in the solvent recovery step, in which separations of mixtures of refined soybean oil/n-hexane, crude soybean oil/n-hexane (industrial miscella) and refined soybean oil/pressurized n-butane were studied. When the commercial ceramic membranes with molecular weight cut-offs between 5 and 10 kDa were investigated, oil rejections up to 100%, total permeate fluxes (oil + solvent) up to 42.97 kg·m^−2^·h^−1^ with oil permeate fluxes up to 1.4 kg·m^−2^·h^−1^ were observed. 

Amelio et al. [52] applied a hybrid process of distillation/pervaporation with the ceramic membrane, hybrid silica (HybSi) and showed that it can give both economic benefit and low environmental impact (with life cycle assessment) in solvent recovery (acetone from water) when compared to conventional waste solvent incineration. Furthermore, this hybrid process will bring an economic benefit of the replacement cost of fresh solvent (about 850 U$S/ton), which is considered as a credit value. Nagasawa [53] applied the same HybSi membranes for the water/ isopropyl alcohol (IPA) separation, in which their water fluxes were like that of NaA zeolite membranes, but their separation factors are lower than NaA zeolite membranes. Clay-alumina-based tubular MF membranes were explored as a viable option for solvent separation and possess advantages such as high micronutrient content (1.56% oryzanol) and negligible oil loss (2.6%) by Roy et al. [54]. When the membranes were operated for 10 h with a 0.7 bar trans-membrane pressure, permeate fluxes of 15 and 8 L/m^2^·h were achieved for the degumming-dewaxing and deacidification operations. Maitlo et al. [55] introduced hydrophobic-oleophilic nature into silica membranes and the membrane showed 100% efficiency for toluene solvent from water within 50 min. All the other membranes tested also showed good efficiency for solvents and no permeability for water, which can be from potential candidates for oil–water and organic solvent–water separation. In recent years, the development of the high-silica zeolite beta (HSZB) membrane has received much attention in the literature due to its high hydrophobicity and excellent mechanical, thermal, and chemical stabilities [56].

**Figure 3:**
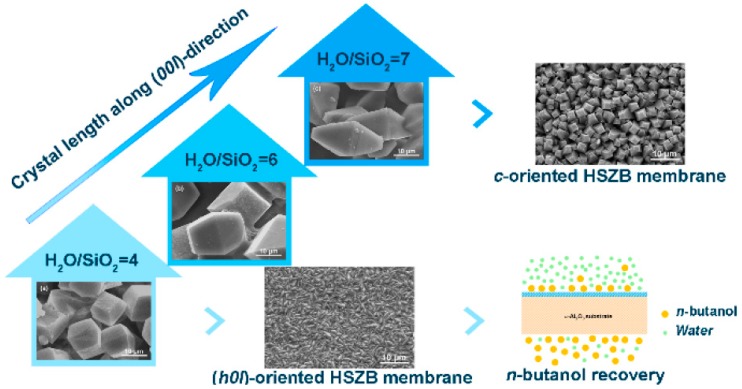
The influence of H_2_O/SiO_2_ ratio on the formation process of HSZB membranes with preferred orientation during an evolutionary growth process. Reproduced with Permission from [57].

Li et al. [57] have fabricated HSZB membranes with controllable orientation (higher H_2_O/SiO_2_ ratio) on randomly oriented seed layers via a secondary growth method. Preferential (h0l)-oriented HSZB membrane was achieved when the H_2_O/SiO_2_ ratio was 4, whereas the c-oriented was obtained when the H_2_O/SiO_2_ ratio of 7 (Figure 3). The (h0l)-oriented HSZB membrane exhibited high fluxes up to 1.45 and 1.05 kg·m^−2^·h^−1^ with respect to 1 and 5 wt% n-butanol/water mixtures, and the corresponding separation factors were 36.5 and 32.5, respectively. This study indicates that (h00l)-oriented HSZB membranes can be prepared at higher H_2_O/SiO_2_ ratio and would be more promising for n-butanol recovery from dilute aqueous solution due to high fluxes with relatively high separation factors because of high hydrophobicity.

Taleb et al. [58] has used natural Moroccan clay for the development of a ceramic support for the purpose of microfiltration. This clay mostly consists of Al_2_O_3_ and SiO_2_, and exhibits properties like high mechanical resistance, high chemical and thermal stability. The macroporous tubular support uses an intermediate layer of ZrO_2_ material to serve the purpose of microfiltration of methylene blue. The supports are fabricated by extrusion process along with heat treatment and then they are coated with dispersed ZrO_2_ and dried. The support displays a permeability of 1926 L/h·m^2^·bar for pure distilled water and a maximum rejection rate of 3.8% for the treatment of methylene blue in the microfiltration process.

### 2.3. Mixed Matrix Membrane

The customization of organic-inorganic polymer hybrids, which involves the combination of properties of both the materials, gives rise to a Mixed Matrix membrane (MMM) or nanocomposite membrane. These membranes consist of two phases: one being the continuous phase acts as the support medium and the other is a filler in a stationary phase. The supporting medium is also known as the matrix. It is usually made of polymers, while the reinforcements provided are in nanoscale dimensions. The addition of nanoparticles to the membranes tends to modify their structure as well as enhance their properties. Incorporation of nanoparticles in the membrane for modification purpose can be done by the following methods: (1) addition of nanoparticles into the polymeric solution before casting, (2) deposition of nanoparticles on the surface of the membrane, and (3) nanoparticles are used to fill the pores on the polymeric membrane.

Livingston et al. [19] prepared a nanocomposite with TiO_2_ nanoparticles incorporated in the crosslinked Polyimide membrane matrix and reported that the addition of reinforcement before the casting process resulted in suppression of macro sized pores on the membrane morphology. The TiO_2_ incorporation also enhances the hydrophilicity and mechanical properties of the system, with the water contact angle decreasing from 81° to 54° and steady ethanol flux settling at 128 L·m^−2^·h^−1^ and 105 L·m^−2^·h^−1^ for DMF. In the case of Organic Solvent Nanofiltration (OSN) membranes, the rejection and flux rates might not be satisfactory due to the pores in nanoscale, but the embedding of gold nanoparticles in cellulose acetate membranes with the absence of defects on the active layer was approached by Vanherck et al. [59], with a 15% increment in the water flux and 400% for pure solvents like ethanol and isopropanol. In this case, the reinforcement present in the membrane was 2 wt% and heat was provided to the membrane via light irradiation. Stronger heat treatment for the composite can be given via laser radiation when the gold nanoparticles are well dispersed in the polymer matrix during in situ synthesis with a mean particle size of 5 nm and larger particles with a maximum size of 20 nm. At high pressures of 3.5, 4.5 and 10 bar, the ethanol flux displayed an average of 0.25 L·m^−2^·h^−1^, 0.4 L·m^−2^·h^−1^ and 1 L·m^−2^·h^−1^, respectively. The authors concluded that the membrane flux is improved by photothermal heating, without a major reduction in the rejection. The gradual increase in intensity of radiation determines the increase in permeance. 

Functionalization of carbon nanotubes has found its application in various fields of research and is capable of enhancing the separation process, due to their unique properties such as high flexibility, low density and existence of substantial nanochannels. The design of amine functionalized multi walled carbon nanotubes (NH_2_-MWCNT) with the matrix of P84 polyimide by Farahani et al. [60] has led to the formation of cross-linked mixed matrix membrane with enhanced flux for organic solvent nanofiltration (OSN) purposes. The hydrophilic carbon nanotubes not only enhance the liquid sorption and transportation in the membrane, but also increase the porosity and pore size, which in turn elevates the solvent fluxes. Although the higher loading of fillers could decline the rejection, they could also lead to agglomeration. The same group also fabricated MMM with the functionalization of MWCNT consisting of carboxyl group in P84 polyimide matrix for OSN applications. The properties such as transfer, sorption and porosity were enhanced with the hydrophilic functional groups on the MWCNT fillers. With the maximum embedding of 0.075 wt% of the filler, the permeance of solvents such as water, ethanol and isopropanol across the membranes increases, as a higher concentration of carbon nanotubes can lead to agglomeration and reduce separation performance. A similar crosslink of MWCNT-COOH has been introduced for the purpose of organic solvent nanofiltration by Farahani et al. [61]. He reported the permeance of solvents like pure water, isopropanol and ethanol, and rejection of rose Bengal in ethanol and isopropanol solutions. The permeance of the solvents depend on the MWCNT-COOH loadings, where the permeance increases with an increase in the content of fillers in the membrane from 0 to 0.05 wt% and the permeance decreases with further increase in the filler loading. However, the cross-linked MMM with loading of 0.05 wt% of MWCNT-COOH results in 99% rejection of rose Bengal in isopropanol and 85% in ethanol with a permeance of 9.6 L/m^2^·h bar at 5 bar.

Defect free layers of poly(dimethyl siloxane) (PDMS) were incorporated with ZIF-8 nanoparticles for the application of permselective pervaporation. Mao et al. [15] stated that the uniform dispersion of ZIF-8 nanoparticles enhanced the hydrophobicity, thermal stability and affinity towards ethanol, which results in selectivity of larger content. The MMM was synthesized by in situ fabrication, where the synthesis time played a vital role in regulating the contact angle of the hydrophobic surface of the membrane. With increase in time from 3–10 min, the contact angle varied from 124° to 138°, but, with variation of time from 10–30 min, there was a decline in the contact angle to 127°. The increase in feed flow rate results in a comparative rise in permeation and separation flux of the membrane. Concurrently, the permeability of water and ethanol was enhanced with feed flow rate; at 40 °C, the MMM separating 5 wt% ethanol aqueous solution displayed a flow rate of 90 Lh^−1^, along with a separation factor of 12.1 due to the defect free active layer and also achieved a high permeation flux of 1778 g·m^−2^·h^−1^. As a result, durable membranes with the ability to perform under different conditions were fabricated.

**Figure 4:**
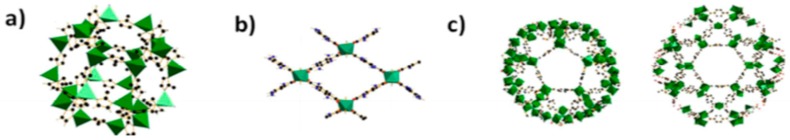
(**a**) Building blocks of ZIF-8 with ZnN_4_; (**b**) pore system in NH_2_-MIL-53 with AlO_6_; (**c**) building blocks of MIL-101 with mesoporous cages; Reproduced with permission from [63].

Metal Organic Frameworks (MOFs) are in affinity with the polymeric chains rather than the inorganic ones due to the presence of organic linkers. The embedding of hydrophobic and hydrophilic MOF nanoparticles (MIL-53-Al, MIL-101-Cr, ZIF-8, and NH_2_-MIL-53(Al), Table 2) into polyamide layer in order to form thin film nanocomposite membranes were reported by Sorribas et al. [62]. Around 0.2 wt% of synthesized MOFs was dispersed in the organic phase to produce thin film nanocomposite membranes (TFN-MOF membrane). ZIF-8 nanoparticles being a hydrophobic MOF displayed an increase in contact angle, while MIL-53 Al, NH_2_-MIL-53(Al) and MIL-101-Cr displayed small contact angle values due to the hydrophilic nature. The changes in hydrophilicity can lead to changes in the chemical structure of the thin film, which can either hydrate or release heat when in contact with organic solvents. The observed permeance results indicate that all the membranes exhibited a rejection greater than 90%, with the permeance varying from 1.5 to 3.9 L·m^−2^·h^−1^·bar^−1^ (Figure 4, Table 3). The order of increase in permeance of methanol/PS (Polystyrene) in the TFN is NH_2_-MIL-53 (Al) < MIL-53(Al) < ZIF-8 < MIL-101(Cr).

Pervaporation can be used for the separation of liquid mixtures when filtration fails to do so. Sorribas et al. [62] discussed that, in the separation of ethanol/water mixture, HKUST-1 hydrophilic MOF is used in 40 wt% mixed membrane matrices. The incorporation of MOF in the membrane matrix increased the flux by two times and left the selectivity and permeability of water unaltered in the membrane. Su et al. [64] presented a high separation performance of alcohol/water distillation when ZIF-8 nanoparticles were incorporated into PDMS hollow fiber membrane modules. The ZIF-8 nanoparticles enhanced the permeability of both isopropanol and water molecules through the membrane due to high surface area and porosity; this eventually increased the mass transfer efficiency between the phases from 0.95 cm/s to 1.65 cm/s. This membrane displayed higher IPA concentration of 62.2% (mol/mol) with height mass transfer unit (HTU) value of 4.9 cm, when compared with the pure PDMS membrane module.

The addition of Mesoporous silica sphere (MSS) with ZIF-71 into PDMS matrix was studied by Naik et al. [65]. The incorporation of composite spheres in PDMS showed relatively improved flux and separation factors for water and ethanol mixtures under pervaporation, in comparison with the pure and MSS filled PDMS membranes. For the separation of 6% aqueous ethanol at 40 ºC for various filler loading (0, 10, 15 and 20 wt%), the normalized flux and separation factor were enhanced by addition of the filler. The reported fluxes and separation factor for the loadings 0, 10, 15 and 20 wt% of the filler are 0.32, 0.91, 0.95 and 1 kg/m^2^·h, and 8, 8.2, 11 and 13 ß, respectively. Similarly, for the addition of ZIF-8 along with MSS in the PDMS matrix, the flux and separation factor displayed values of 0.63 kg/m^2^·h and 14 ß, respectively, for the loading of 20 wt% of the filler.

Fan et al. [66] studied the fabrication of ZIF-8-PDMS membrane for the recovery of n-butanol from aqueous solution. The ZIF-8 nanoparticles were added to the membrane surface through the process of simultaneous spray self-assembly. At extremely high loading of 40 wt% of ZIF-8 in PDMS, high total flux of 4846.2 kg/m^2^·h and separation factor of 81.6 were obtained at a feed temperature of 80 °C. The flux and separation factor values attained for pure PDMS are 1000 kg/m^2^·h and 38 and tend to increase with addition of ZIF-8 up to 40 wt%.

### 2.4. Metal Organic Frameworks

Metal organic frameworks (MOF) are hybrid crystalline materials having metal ions linked with a network of organic ligands. They have a porous structure due to the presence of potential voids in the organic network and have large surface area with new topologies. These pores serve the purpose of storing molecules and the structure might experience instability during the elimination of molecules. These functional materials have found various applications such as ion exchange, adsorption and catalysis. The organic solvent nanofiltration (OSN) membranes face a major challenge in establishing materials that are stable and suitable for producing high performance over a long span of time, as the early MOFs displayed poor chemical stability with atmospheric moisture along with easy deterioration of framework. On overcoming their disadvantages, MOFs presented a structural diversity greater than that of zeolite and exhibited surface areas of 7000 m^2^·g^−1^. The membrane performance can be varied based on the post treatments conditions as well as the MOF loadings. MOFs have pore size ranging from 0.3 nm to 10 nm depending on composition and positioning MOF membranes in the nanofiltration establishment. The membranes involved in the organic solvent nanofiltration are evaluated based on the permeance and rejection of chemical compounds. The selective permeability in MOF membranes can be achieved by molecular sieving, and the presence of high porosity within them favors the permeates’ high flux with a high rejection of contaminants. The integration of selective pores and molecular sieving concept in the membranes incorporates both the desirable properties required for the membranes like, high selectivity and flux. In the case of ZIF-78, Kasik et al. reported [67] the separation of cyclohexanone–cyclohexanol mixture through pervaporation, with 50:50 ratios experiencing a permselectivity of 1:2 and a total flux of around 8.7 × 10^−2^ kg·m^−2^·h^−1^ at room temperature.

#### 2.4.1. MIL-101 (Cr)

MIL (Materials Institute Lavoisier) are MOFs with chromium as the metal linking to organic linkers. Sorribas et al. [63] loaded different concentrations of MIL-101-Cr in thin film nanocomposite, and noticed that the permeance and rejection varied generally. The concentration was varied between 0.05–0.4 wt%, and the permeance of tetrahydrofuran and methanol increased gradually up to 0.2 wt% loading. Nonetheless, there was a slight increase in permeance from 0.2 to 0.4 wt% as a result of aggregation of particles at high concentration, and not many changes were observed for the rejection on the basis of MOF loading. The nanoparticles embedded a TFN membrane with 0.2 wt%. MOF displayed an increase of 160% permeance compared to the thin film composite in the case of methanol/PS with 3.9 L·m^−2^·h^−1^·bar^−1^ (from 1.5 L·m^−2^·h^−1^·bar^−1^), whereas the tetrahydrofuran (THF)/PS permeance increased to 488% with permeance up to 10.0 L·m^−2^·h^−1^·bar^−1^ (1.7 L m^−2^·h^−1^·bar^−1^), which is due to low viscosity of THF. For the loading of 0.4 wt% of MIL-101 (Cr), the permeance was reported as 11 L·m^−2^·h^−1^·bar^−1^ with a rejection of THF greater than 90%. This TFNC achieved high performance in organic solvent nanofiltration with increasing permeance and preserving the high rejection.

#### 2.4.2. MIL-53

Materials Institute Lavoisier is a metal organic framework based on aluminium that is linked with carboxylate groups into a three-dimensional network. These flexible MOFs are capable of altering their shape and size with heat treatment and adsorption of CO_2_. Van der Bruggen et al. [68] conducted a study on the incorporation of MIL-53 (Al) into a polymer matrix to perform the filtration of organic solvents with pore size distribution between 0.4 nm to 1.1 nm, and they obtained results with increased permeance of solvents like ethanol. MIL-53 was embedded into the polyamide matrix with different concentrations of the filler (0.3, 0.5, 1.0 and 1.5 wt%). The membranes displayed fluctuations on exposure to the organic solvents. In the case of the membrane with 0.5 wt% concentration of the filler, the membrane observed stability was 10 days, and also showed the increase in ethanol permeance from 0.2 L·m^−2^·h^−1^·bar^−1^ to 0.7 L·m^−2^·h^−1^·bar^−1^ (up to 289%) and a slight reduction in the brilliant blue rejection from 97% to 94% (3% less). Zhu et al. [68] reported the application of MIL-53 for the dehydration of water–ethyl acetate mixtures through pervaporation at a temperature of 60 °C, where the high water selectivity led to the permeate containing 99% water and the 7% feed. Furthermore, the membrane with the fillers exhibited high stability of 200 h of operation. The flux of the membrane rises with increase in the MOF content (0–0.5 wt%), and the increase in flux is a result of the hydrophilicity of MIL-53 (Al) (Table 4). Increased inclusion of MIL-53(Al) particles has resulted in aggregation of them and, in turn, decreased the flux of the membrane. Decrease in the contact angle was observed for the membranes due to hydrophilicity of the added MIL-53, which has increased the affinity of the matrix to water molecules.

**Figure 5:**
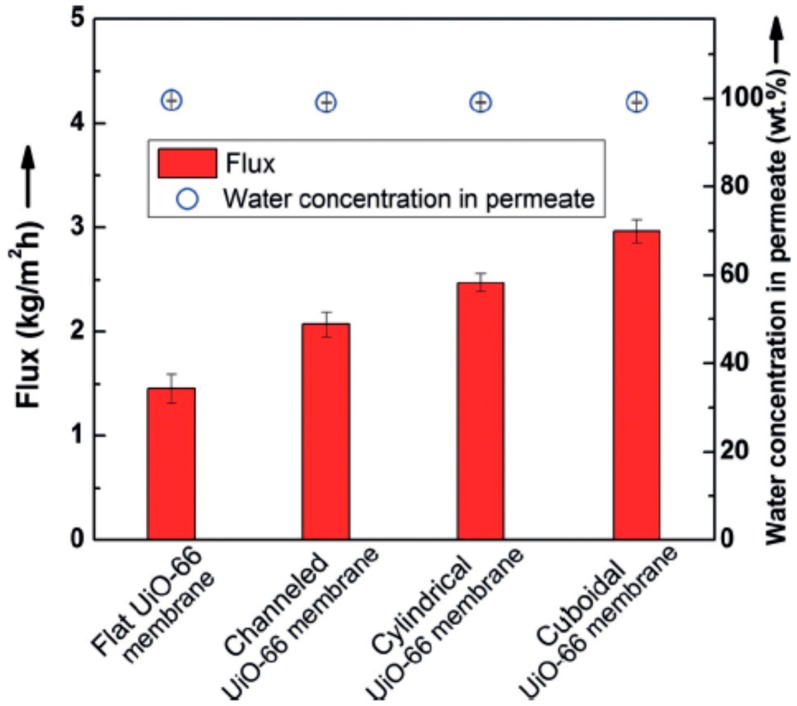
Separation performance of UiO-66 membranes with different patterns; Reproduced with permission from [69].

Li et al. [69] applied the micropatterning concept for the first time to MOF membrane with enhanced molecular sieving property, which can used in compact devices. Ultrathin polycrystalline zirconium-MOF UiO-66 membranes with a thickness down to 250 nm on a patterned porous yttria-stabilized zirconia (YSZ) ceramic substrates were grown by a bottom-up procedure. The patterned UiO-66 membranes exhibited good molecular separation property (separation factor of over 1000) and 100% improvement in the apparent permeation flux of 2.96 kg·m^−2^·h^−1^ (patterned UiO-66 membranes were considerably higher than the unpatterned membrane, Figure 5) in butanol dehydration through the pervaporation process and showed that growing high-quality MOF thin films (defect-free) on complex surfaces can be achieved in MOF membrane formation. 

In MOF, organic linkers are attached with metal ions that are sufficiently strong to make the MOF structure adequately robust and uniform distribution of metal active sites. Zirehpour et al. [70] immobilized MOF nanocrystals into the active layer of the forward osmosis (FO) membranes via dispersion in the organic solution followed by interfacial polymerization. The immobilization improved biofouling resistance in the membranes, with 8% decline in flux after 24 h of operation in biofouling experiments, whereas control membranes had a greater decline of ~21%. A similar approach can be applied in MOF membranes in the solvent recovery applications, as membrane antifouling behaviour will improve membrane performance, which will decrease operational costs, and will also increase the membrane life. 

Although MOFs are synthesized in the form of large crystals or micro-crystalline powders, they have few drawbacks such as inferior mechanical properties (fragile, brittle) and low processability, thereby hindering their utilization of defect-free polycrystalline membranes for industrial scale applications. Satheeshkumar et al. [71] have overcome these issues, by the fabrication of a free-standing mixed-matrix membrane (MMM) containing covalently incorporated metal–organic framework (MOF) particles (up to 60 wt%) consisting of vinyl functionality by applying the thiol-ene photopolymerization technique. The free standing MMM was readily produced by irradiation of a polymerization mixture containing UiO-66-CH=CH_2_ (synthesised from 2-vinyl-1,4-dicarboxylic acid with ZrCl_4_) poly(ethylene glycol) divinyl ether (PEO-250), pentaerythritol tetra(3-mercaptopropionate) (PETM), 2,20-(ethylenedioxy)diethanethiol (EDDT), and 2,2-dimethoxy-2-phenylacetophenone (DMPA) as a photoradical initiator. 

Although studies on MOF films on various copper supports such as plates, disks, meshes, beads and copper coated silicon wafers, using gold and glass carbon electrodes, were reported, electrochemical deposition of MOFs directly on porous hollow fibers (HFs) have not been reported. Demirel et al. [72] reported on the thin metal organic framework (Cu-BTC, (Cu_3_(BTC)_2_,BTC =benzene-1,3,5-tricarboxylate) films on Cu-HFs, to study the effect of the presence of a supporting electrolyte and the magnitude of the applied electrical potential on the formation and the morphology of the films. In the presence of a supporting electrolyte, formation of less homogeneous films and the growth of MOF crystals were observed in the liquid, whereas, in the absence of a supporting electrolyte, and at low potential, more uniform films with smaller particles were obtained. 

#### 2.4.3. ZIF 

(1) ZIF-8

Zeolite imidazolate framework (ZIF) is a metal organic framework consisting of four imidazolate rings linked to zinc ions. The metal ions are covalently bridged with imidazolate in a similar pattern to silicon or aluminum with oxygen in zeolites. ZIF-8 has a porous structure with pores in micro to nanometer range and topology that is indistinguishable to zeolite. ZIFs exhibit properties like high surface area, robust porosity, thermal and chemical stability, and high specificity. Studies have showed their potential in separation of hydrocarbon mixtures like ethane/propane, ethylene/propylene and alcohols. They tend to offer high separation factor at a low energy consumption. The hydrophobic nature makes it difficult for the extraction of pollutants in an aqueous phase due to the poor contact between phases. Therefore, Maya et al. [73] engineered dispersions of ZIF-8 crystals in binary solvent mixtures for the extraction of solvents, identical to the dispersive liquid-phase microextraction technique. However, ZIF-8, with hydrophobic pore surface and hydrophilic crystal surface, along with aperture size of ~3.4 Å seems to be unfavorable for the transfer of organic molecules. Xu et al. [74] incorporated β-CD@ZIF-8 into various polymer supports, and tested these membranes for their dye/solvent separation performances. The presence of ZIF in the selective layer enhances the permeance of solvent. In the separation of rose Bengal and the solvent using PA membranes with β-CD@ZIF-8 nanoparticles embedded in them, they attained rejection values of 96.2 ± 1.6% of RB/MeOH, and 95.0 ± 1.1% of RB/THF. Similarly, for PPA-05matrix, -β-CD@ZIF-8 rejection of 96.6 ± 1.8% of RB/MeOH and 94.5 ± 0.5% of RB/THF were observed. With the addition of 25 wt% β-CD@ZIF-8 in PA, the water contact angle was minimized, which reduced the resistance to mass transfer of solvents and also resulted in the highest methanol flux in MF (mass flux) scale. ZIF-68 membranes with large pores were applied for the molecular sieving of large sized liquid molecules. Kasik et al. [18] reported the pervaporation of p-xylene using ZIF-68, synthesized by reactive seed method, which produced flux of 492 × 10^−5^ mol/m^2^·s, 5.4 times greater than MOF-5 membranes. However, pervaporation flux of the larger size molecules using ZIF-68 membranes was lower than MOF-5. 

(2) ZIF-90

**Figure 6:**
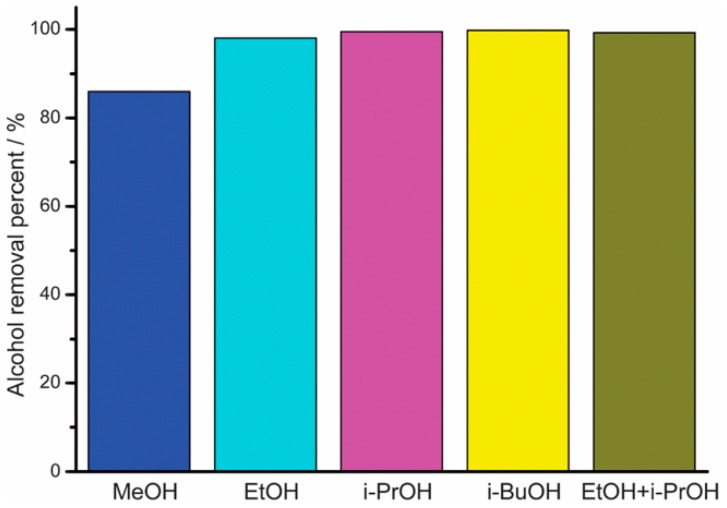
Adsorptive separation performance from of the superhydrophobic ZIF-90 for the removal of alcohols from alcohol/water mixtures; Reproduced with permission from [75].

MOFs with linkers of transition metals and imidazolate have porous structure, making them desirable candidates for molecular sieve membranes. The potential to adjust the pore size and surface properties of the ZIF has found its application in the recovery of bio-alcohols by using them as adsorbents. Liu et al. [75] reported the amine aggregate functionalisation on ZIF-90 by covalently connecting with free aldehyde functional groups present in the structure through host–guest interaction. Liu et al. reported the combination of a superhydrophobic ZIF-90 for the purpose of bioalcohol recuperation through the post-functionalization of ZIF-90 with penta-fluorobenzyl amine by means of an amine group. It is generally presumed that the fluorinated ZIF-90 will display a high hydrophobicity and, in this way, it is a promising candidate for bioalcohol recuperation. The reaction between pentaflurobenzylamine and ZIF-90 takes place on the external surface of ZIF-90 through the condensation of amine groups. The surface of the fluorinated ZIF-90 showed a water contact angle of about 152.41; while the contact angle of water on the surface of the as-arranged ZIF-90 is 93.91°, it indicates that the hydrophobicity of the ZIF-90 can be increased through post-functionalization with pentafluorobenzyl amine. About 98% ethanol was removed from the mixture of ethanol/water from the superhydrophobic ZIF-90 (Figure 6). In contrast, only 7% ethanol can be removed while applying as-prepared ZIF-90 as the adsorbent. Besides ethanol, the superhydrophobic ZIF-90 displays high adsorptive separation performances for the other bio-alcohols such as methanol, iso-propanol and butanol, along with its mixtures.

(3) ZIF-71

Yin et al. [13] reported the recovery of solvents like ethanol and 1-butanol using ZIF-71/PDMS nanocomposite membranes. Being a hydrophobic polymer with the ability to perform the pervaporation process, polydimethylsiloxane (PDMS) was embedded with different quantities of ZIF-71 fillers. The content of fillers was optimized for 40 wt% load of ZIF-71 in PDMS attaining the maximum selectivity of 0.81 ± 0.04 (separation factor of 12.5 ± 0.3 for 2 wt% ethanol feed) and maximum 1-butanol/water selectivity of 5.64 ± 0.15 (separation factor of 69.9 ± 1.8 for 2 wt% 1-butanol feed). The composites displayed a decrease in fracture strain from 381.4 to 35.6 J/cm^2^ when formed through a condensation cure rather than an addition cure. Membranes with 40 wt% ZIF-71exhibited maximum permeability and selectivity, for ethanol/water mixture, with ethanol permeability of 24,809 ± 4374 Barrer, water permeability of 30,661 ± 4015 Barrer and selectivity of 0.81 ± 0.04. For 1-butanol/water separation, 40 wt% ZIF-71 loading MMMs had the highest 1-butanol permeability of 123,045 ± 17,118 Barrer, water permeability of 21,758 ± 2497 Barrer and selectivity of 5.64 ± 0.15.

**Figure 7:**
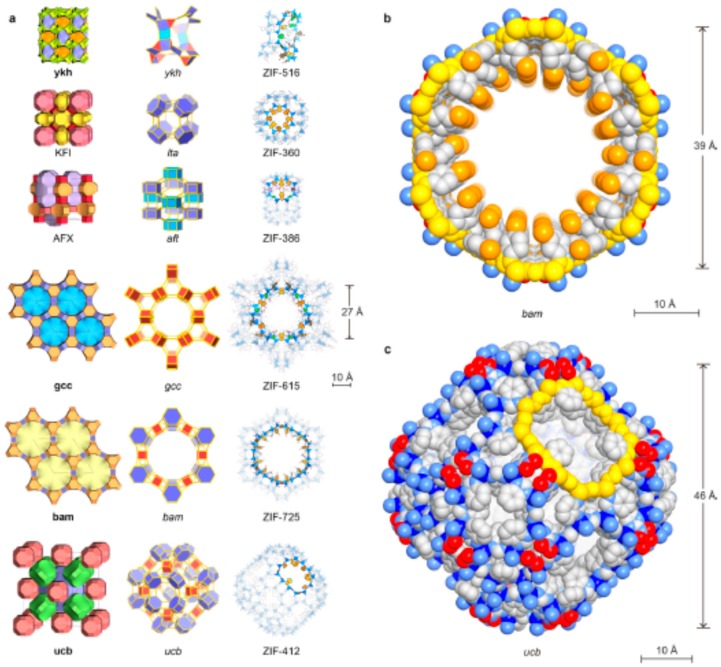
Crystal structures of the new ZIFs. Topologies are shown in natural tilings. The largest cages are presented with adjacent small cages, and characteristic cages are shown with ball-and-stick structures for linkers (N, blue; C, dark; O, red; Cl, green; Br, orange, H, omitted for clarity) and blue tetrahedra for ZnN4 units. (**a**) KFI, ZIF-360; AFX, ZIF-386; ykh, ZIF-516; gcc, ZIF-615; bam, ZIF-725; ucb, ZIF-412. Largest openings for each cage are highlighted. (**b**) Space-filling views for the channel in bam ZIF (ZIF-725) are shown (zinc, blue; N, light blue; C, gray; O, red; Br, orange). The 24-MR aperture (bam ZIF-725, 96 atoms) is highlighted in yellow. (**c**) Space-filling view for the largest cage in ucb ZIFs (illustrated by ZIF-412) is shown: zinc, blue; N, light blue; C, gray; O, red. The 12 MR opening (ucb ZIF-412, 48 atoms) is highlighted in yellow. Reproduced with permission from [7].

In addition to the above ZIFs, several other ZIF materials with increased pore sizes were synthesized [7] and tested for various applications (Figure 7). Some of the notable ZIF materials pore sizes are reported below in Table 5. It is to be noted here that ZIF membranes of smaller pore ZIF are synthesized extensively and studied for the separation of smaller molecules, such as H_2_ purification and CO_2_ sequestration. On the other hand, few research studies were focused on the synthesis of larger pore ZIFs and investigated for the separation of large size gas molecules and liquid separations (by adsorption mechanism).

##### Challenges in the Formation of ZIF Membranes

In the recovery of solvent mixtures, the excellent properties of ZIF such as pore size, chemical stability, thermal stability and structural stability have motivated the researchers to explore their industrial application. During the usage for these membranes for industrial applications, they show poor coverage of ZIF on the support membranes due to the growth of large sized ZIF crystals [19]. To overcome this obstacle, researchers have concluded that ZnO coating is ideal for the modification of membrane to coat ZIFs [80].

In the synthesis of high quality ZIF-71 crystals using zinc acetate and 4,5-dichloroimidazole (dcIm) as precursors in methanol, the organic linkers were consumed instantly upon their introduction to the zinc ions and produce the crystals of ZIF-71 in the solvent medium rather than on the porous support. The secondary growth for the preparation of ZIF-71 membrane in methanol was observed to be unfavorable by Dong et al. [80] with a contrast of relatively slow and controllable reaction rates of the precursors in DMF solvent, due to the difference in deprotonation rates of dcIm in the two solvents (Table 6). The high affinity of organic molecules to the membrane surface and channel are the vital roles in the segregation of organic solvents with membrane pore size larger than the molecular size of solvents. ZIF-71 consists of organophilic surfaces on both the sides and can act as desirable membrane material for the separation of organic molecules via pervaporation. Separation studies show that the ZIF-71 membrane displays lower adsorption performance than that of the ZIF-71 crystals due to relatively slow diffusion of solvents. In the case of ethanol, slow diffusion is observed due to the kinetic diameter of 4.53 Å, which is nearly the size of ZIF-71 (4.8 Å). Organic-organic mixtures can be separated through a hydrophobic membrane, where the weak-polar organic molecules show greater affinity towards the membrane than strong polar molecules. In addition, dimethyl carbonate (DMC) molecules with a size larger than ZIF-71 can diffuse through membranes similar to ZIF crystals via flexible frameworks or gate opening effect and showed total flux and separation factor of 271 g·m^−1^·h^−1^ and 5.34, respectively, for 5 wt% dimethyl carbonate–methanol separation. 

**Figure 8:**
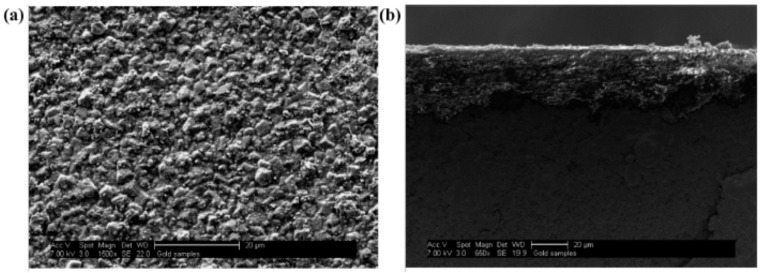
ZIF-68 membrane synthesis step following in situ growth of ZnO on Al_2_O_3_ SEM micrographs showing ZIF-68 membrane layer formed following secondary growth at (**a**) 1500× and (**b**) a cross-sectional picture at 660×; Reproduced with permission from [18].

**Figure 9:**
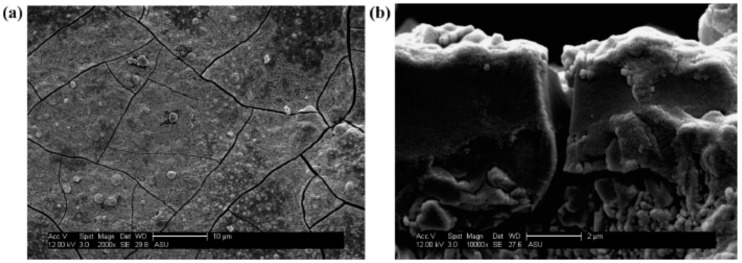
ZIF-68 membrane synthesis step following dip-coating of Al_2_O_3_ support in ZnO. SEM micrographs of ZIF-68 membrane layer on Al_2_O_3_ support following dip coating of ZnO, reactive seeding, and secondary growth at (**a**) 2000× and (**b**) a cross-sectional view at 10,000×; Reproduced with permission from [18].

Kasik et al. [18] reported that secondary growth of ZIF-68 with continuous layer can be synthesized on the alumina support modified by ZnO via an in situ method (Figure 8) when compared to the ZnO via dip coating (Figure 9).

Li et al. [81] have overcome the inferior processability of the MOFs (such as ZIF) and scalability, which hinders their application in various domains, by gel coating for precursor dispersion followed by thermal-treatment for crystallization. This is similar to the processing of polymeric membranes that can be applied with controllable thickness on various substrates. The asynchronous crystallization between the bottom and the top of gels that found was critical for the formation of defect-free MOF (such as ZIF-8) membranes. Although prepared ZIF-8 membranes with a ligand/zinc ratio of 4 showed impressive performance in gas separation, they have not been utilized for solvent separation, which can be explored in the future. 

Kong et al. [82] applied the rubbing method to coat ZnO particles onto the porous α-Al_2_O_3_ support, which could serve three major functions such as modification of support, induction (as active seed for homogeneous nucleation) and anchoring of ZIF during membrane synthesis. The formed membranes were continuous, uniform and showed good molecular sieve performance of various gases and the method is simple, convenient and scalable. 

#### 2.4.4. Metal Azolate Framework (MAF-6)

MAF-6 a hydrophobic MOF with larger pore and aperture size (~7.6 Å) was introduced by Li et al. [14] The nanoporous zeolitic metal azolate framework, RHO-[Zn(eim)_2_], has the ability to absorb organic solvents like ethanol instantly but is resistant to water. MAF-6 nanoparticles are synthesized at room temperature rather than using high temperature and pressure like most MOFs. The synthesis procedure is highly beneficial because of the high efficiency and low energy consumption. Embedding of MAF-6 nanoparticles in PDMS matrix to produce MMM for the ethanol/water pervaporation separation has resulted in high performance with a separation flux of 1200 g/m^2^·h and separation factor of 14.9, which happens to be 1.5 and 2.3 times of PDMS pristine membrane, respectively. This MAF-6 showed a long-term stability of 120 h, proving their durability.

### 2.5. Grignard Functionalized Ceramic Membranes

Grignard reaction is an organometallic chemical reaction, where alkyl halides are added to carbonyl groups in ketone or aldehyde. Hosseinabadi et al. [83] discussed the use of membranes modified with alkyl groups in them as a prospective membrane for organic solvent nanofiltration. The change in affinity of solvents and solutes for the modified membranes varies the retention of them. The membranes were fabricated using TiO_2_ nanoparticles, which were then functionalized by grafting alkyl groups, using an organometallic Grignard reagent. A solvent with less affinity towards the membrane was chosen to obtain relatively low retentions. For example, toluene is the solvent with lower solvent-membrane affinity in the mixture of toluene/polystyrene. When the mixture was passed down through the Grignard modified membrane, there is an increase in the hydrophobicity of the membrane which also happened to raise the retention of polystyrene in toluene, whereas the retention was increased to 55% from 30% for polystyrene with 580 Da in toluene.

## 3. Pervaporation

**Figure 10:**
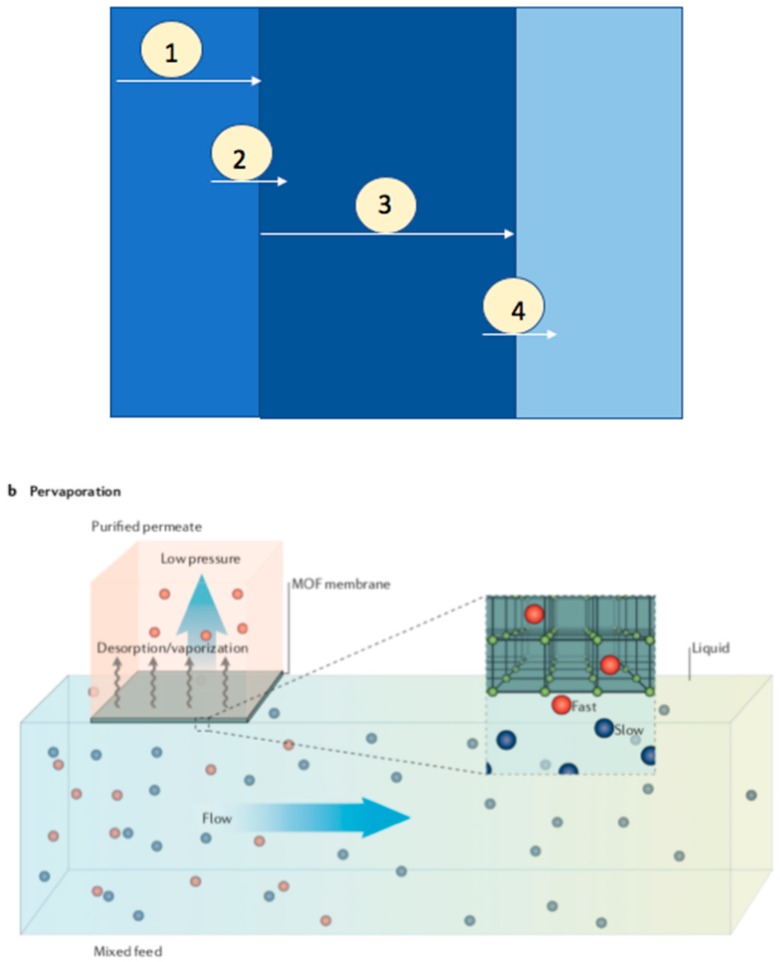
Schematic illustrations for pervaporation method; Reproduced with permission from [18].

The transportation of components in a pervaporation process across the membrane reported by solution desorption model consists of a series of processes (Figure 10) [9]: 1. Diffusion of component to the membrane surface through the liquid boundary layer, 2. Sorption into the membrane, 3. Transportation though the membrane, and 4. Diffusion through vapor phase boundary layer into the bulk of the permeance. The transportation of gas, vapor, or liquid through a membrane is given by permeability, which is the product of diffusivity and solubility. The efficacy of a pervaporation is majorly determined by the separation factor and the permeate flux. The permeability is dependent on the mass divided by both membrane area and time. The product of diffusion coefficient and sorption coefficient equals the flux of dilute solutions. The membrane performance during pervaporation also depends on the following parameters: membrane thickness, temperature and feed concentration. 

## 4. Merits of Solvent Recovery 

(I) Economic Benefit

The process of solvent recycling and recovery is schemed to favor the industrial facility with reduction in liquid waste disposal expenses and recycling of solvent wastes to generate solvents. This helps in bringing down the purchases of expensive solvents [1,8].

(II) Environmental Benefit

In addition to the reduction in investment for solvents and waste through solvent recycling, it also influences the environment. The elimination of hazardous wastes through recycling and recovery reduces the quantity of waste disposed. The reduction in cost of disposal and usage of minimum storage space reduces the impact of the waste on the environment [8].

(III) Regulatory Conformity

According to the Hazardous Waste regulations enforced in the United Kingdom in 2005, any organization producing hazardous waste of more than 500 kg is considered an offence. The aim of these regulations is to prevent pollution and protect the environment and human health [8].

## 5. Conclusions

In the field of solvent recovery, the desire to preserve the solvents from the industry after use has led to the development of various materials chemistry and separation processes. Some of the materials explored are inorganic membranes for the process of solvent recovery and recycling in the industrial scale. On a commercial scale, the membranes seem to be dominated by porous ceramic membranes with shapes like disks, tubes or multi-channel monoliths. These membranes are feasible and commercially sold by Mitsui Engineering and Shipbuilding Company, City, Japan and a few other companies around the world.

These inorganic membranes consist of layers of various materials such as alumina, zirconia, stainless steel and other ceramics. The polymers modified with the embedding of nanomaterials have high permeability and selectivity of solvents. By varying the amount of nanomaterials in the matrix, the nanocomposites properties such as the permeability, selectivity and rejection of solvents are varied. Generally, films made up of zeolites, ZIFs on substrate can be fabricated using in situ synthesis and secondary growth of a reactive seed with hydrothermal treatment. It has been observed that the secondary growth method favors high reproducibility and good control over the membrane structure [18,45]. Recently, the patterned MOF membranes were developed with good molecular separation property and improvement in the apparent permeation flux, which can be applied in compact devices application.

Only very few reports were available on the recovery of solvent mixtures using ZIF materials, which is due to considerable challenges connected with suitable ZIF materials screening and well-connected ZIF membranes formation on support material. This particular area can be exploited further in future.

This paper demonstrates the performances of various Mixed Metal Membranes, MOFs and ceramic membranes in the solvent recovery process with respect to permeability, selectivity and rejection flux.

## Figures and Tables

**Table 1:** Pervaporation (PV) performance of the zeolite NaA membranes toward water/ organic liquid systems; Reproduced with permission from [24].

Separation System	Temp (K)	X_W_ (wt%)	Q (kg/(m^2^·h))	α [-]
Ethanol	348	10	2.15	10,000
	348	5	1.10	16,000
	348	0.5	0.012	5100
Methanol	323	10	0.57	2100
	323	5	0.23	2500
n-propanol	348	10	1.91	18,000
i-propanol	348	10	1.76	10,000
Acetone	323	10	0.91	5600
	323	5	0.83	6800
Dioxane	333	10	1.87	>9000
DMF	333	10	0.95	>9000

Temp—Temperature; Xw—Weight percentage (wt%); Q—Total Permeation Flux; α—Separation factor.

**Table 2:** Contact angle of thin film composite (TFC) and thin film nanocomposite MOF (TFN-MOF) membranes with 0.2% (w/v) concentration in the organic phase before Interfacial polymerization (IP) reaction. For TFN-MIL-101, concentration was changed from 0.05 to 0.4% (w/v); Reproduced with permission from [62].

Membrane	Contact Angle (°)			
TFC (without MOF)	-	-	~73	-
TFN-NH_2_-MIL-53 (Al)	-	-	~49	-
TFN-MIL-53 (Al)	-	-	~54	-
TFN-ZIF-8	-	-	~75	-
TFN-MIL-101 (Cr)	~53 (0.05)	~52 (0.1)	~50 (0.2)	~43 (0.4)

The values in parentheses are subjected to MOF loading in %.

**Table 3:** Organic Solvent nanofiltration (OSN) results of TFN membranes for MeOH at 30 °C and 30 bar. (The values given are an average of 3–4 different membranes); Reproduced with permission from [63].

Membrane	Permeance Methanol (L·m^−2^·h^−1^·bar^−1^)	Permeance Methanol/PS (Polystyrene) (L·m^−2^·h^−1^·bar^−1^)
TFC (without MOF)	1.8	1.5
TFN-NH_2_-MIL-53 (Al)	2.3	1.8
TFN-MIL-53 (Al)	2.3	1.9
TFN-ZIF-8	2.5	2.1
TFN-MIL-101 (Cr)	4.2	3.9

**Table 4:** Performance of membranes with different MIL-53 (Al) contents; Reproduced with permission from Ref [68].

MOF Content (wt%)	Contact Angle (°C)
0	86 ± 2
0.3	79 ± 2
0.5	76 ± 1
1.0	73 ± 1
1.5	71 ± 2

**Table 5:** Preparation and pore sizes of different ZIFs reported in the literature [7].

Material	Molecular Name	Pore Size (Å)	References
ZIF-7	Zn (benzimidazole)_2_	3.0	[16,76,77]
ZIF-8	Zn(2-methylimidazole)_2_	3.4	[16,76,77]
ZIF-90	Zn(imidazolate-2-carboxaldehyde)	3.5	[16,76,77]
ZIF-71	Zn(4,5-dichloroimidazole)_2_	4.2	[16,76,77]
ZIF-69	Zn(5chlorobenzimidazole)(2-nitroimidazole)	4.4	[16,76,77]
ZIF 68	Zn(benzimidazole)(2-nitroimidazole)	7.5	[18]
ZIF 22	Zn(5-azabenzimidazolate)_2_	0.44	[77]
ZIF 78	Zn(5-nitrobenzimidazole)(2-nitroimidazole)	0.38	[78]
ZIF 95	Zn(5-chlorobenzimidazole)_2_	0.37	[79]
ZIF 360	Zn(bIM)_1.00_ (nIM)_0.70_(IM)_0.30_	4.8	[7]
ZIF 365	Zn(cbIM)_0.95_ (nIM)_0.60_(IM)_0.45_	5.0	[7]
ZIF-410	Zn(cbIM)_1.0_ (aIM)_0.90_	5.0	[7]
ZIF486	Zn(nbIM)_0.20_ (mIM)_0.65_(IM)_1.15_	6.0	[7]
ZIF412	Zn(bIM)_1.13_ (nIM)_0.62_(IM)_0.25_	8.2	[7]
ZIF413	Zn(mIM)_1.03_ (nIM)_0.64_(IM)_0.33_	6.8	[7]
ZIF414	Zn(nbIM)_0.921_ (mIM)_0.62_(IM)_0.47_	4.6	[7]
ZIF725	Zn(bbIM)_1.35_ (nIM)_0.40_(IM)_0.25_	22.5	[7]

List of abbreviations used in the above table. bIM- benzimidazole; nIM -nitroimidazole; IM- Imidazole; cbIM- 5-chlorobenzimidazole; aIM- Imidazole-2-carboxaldehyde; nbIM-6-nitrobenzimidazole; mIM -2-methylimidazole; bbIM - 6-bromobenzimidazole.

**Table 6:** Pervaporation performance of the ZIF-71 membrane in the separation of 5 wt% ethanol-water, methanol-water and DMC-methanol at 25 °C; Reproduced with permission from [80].

System	Total Flux (g·m^−2^·h^−1^)	Separation Factor	Alcohol or DMC Permeance (g·m^−2^·h^−1^·kPa^−1^)	Selectivity
EtOH-water	322.18	6.09	117.43	1.50
MeOH-water	394.64	21.38	260.22	4.32
DMC-MeOH	271.21	5.34	102.89	8.08
